# ZnO-Based Ultraviolet Photodetectors

**DOI:** 10.3390/s100908604

**Published:** 2010-09-17

**Authors:** Kewei Liu, Makoto Sakurai, Masakazu Aono

**Affiliations:** International Center for Materials Nanoarchitectonics (MANA), National Institute for Materials Science (NIMS), Tsukuba 305-0044, Japan

**Keywords:** ZnO, photodetector, MSM, *p-n* junction, Schottky, response

## Abstract

Ultraviolet (UV) photodetection has drawn a great deal of attention in recent years due to a wide range of civil and military applications. Because of its wide band gap, low cost, strong radiation hardness and high chemical stability, ZnO are regarded as one of the most promising candidates for UV photodetectors. Additionally, doping in ZnO with Mg elements can adjust the bandgap largely and make it feasible to prepare UV photodetectors with different cut-off wavelengths. ZnO-based photoconductors, Schottky photodiodes, metal–semiconductor–metal photodiodes and p–n junction photodetectors have been developed. In this work, it mainly focuses on the ZnO and ZnMgO films photodetectors. We analyze the performance of ZnO-based photodetectors, discussing recent achievements, and comparing the characteristics of the various photodetector structures developed to date.

## 1. Introduction

Ultraviolet (UV) photodetectors have been widely used in various commercial and military applications, such as secure space-to-space communications, pollution monitoring, water sterilization, flame sensing and early missile plume detection, *etc.* [1]. All these applications require very sensitive devices with high signal-to-noise ratio and high response speed. A variety of UV detectors are available, mainly Si-based photodetectors and photomultipliers. These devices can be very sensitive in UV region with low noise and quick response. However, they have significant limitations, such as the need of filters to stop low energy photons (visible and IR light), their degradation and lower efficiency (Si-based photodetectors), or the need of an ultra-high vacuum environment and a very high voltage supply (photomultipliers) [2]. To avoid these disadvantages, UV detectors based on wide bandgap semiconductors (such as diamond, SiC, III-nitrides and wide-bandgap II–VI materials) have received more and more attention due to their intrinsic visible-blindness. Moreover, wide-bandgap materials are chemically and thermally more stable, which is an advantage for devices operating in harsh environments [3].

Among them, ZnO has been studied extensively in recent years for their unique properties and potential applications of electronic and optoelectronic devices [4,5]. It has strong radiation hardness, high chemical stability, low cost, and a large bandgap of 3.37 eV at room temperature [6,7]. Furthermore, doping in ZnO with Mg elements can adjust the bandgap and make it feasible to prepare UV photodetectors with different cut-off wavelengths [4,8].

The UV photoresponse in ZnO films was first observed by Mollow in the 1940s [9]. However, the research of ZnO based photodetectors flourished gradually since the 1980s [10]. At the beginning, the devices usually have simple structure and the properties are not very good. With improvement of the fabrication of the ZnO-based films using different techniques, many complex ZnO-based photodetectors (such as *p-n* junction, *p-i-n* junction and Schottky junction, etc.) with high performance were reported.

In this paper, we review the recent progress in ZnO-based photodetectors. This paper focused on ZnO-based thin film devices, the nanostructure versions have been discussed in recent review articles [11,12] and are not repeated here. The organization of this review is as follows: First, ZnO photodetectors including photoconductors, metal-semiconductor-metal (MSM) photodetectors, Schottky photodiodes, *p-n* junction photodiodes are discussed in Section 2. This is followed by ZnMgO photodetectors in Section 3. Finally, we conclude this review with some perspectives/outlook and future research directions in this field.

## 2. ZnO-Based Photodetectors

In this section, ZnO-based photodetectors, inclusive of photoconductors, metal-semiconductor-metal (MSM) photodetectors, Schottky photodiodes, *p-n* junction photodiodes are discussed.

### 2.1. Photoconductors

**Figure 1.**
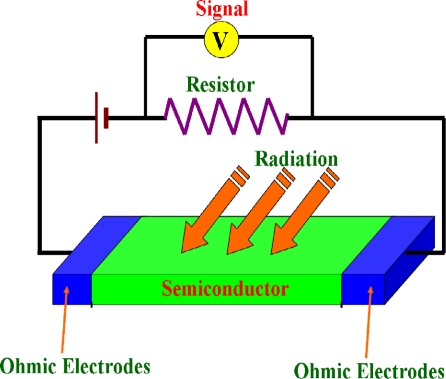
The schematic structure of photoconductors.

A photoconductor, consisting of a semiconductor with two ohmic contacts, is essentially a radiation-sensitive resistor [13]. The schematic structure and operation of the photoconductor are shown in Figure 1. Usually, the load resistance is much smaller than the device resistance. When a photon with the energy larger than the band-gap energy of semiconductor is absorbed, an electron-hole pair would be produced, thereby changing the electrical conductivity of the semiconductor.

Photoconductors present high internal gain at room temperature, so they have large photoresponsivity and can work without any amplifying equipment. However, this gain is associated with a sublinear behaviour with incident power, poor UV/visible contrast, and persistent photoconductivity effects. Therefore, for a practical application, we must think about internal gain and other parameters according to our need.

**Figure 2.**
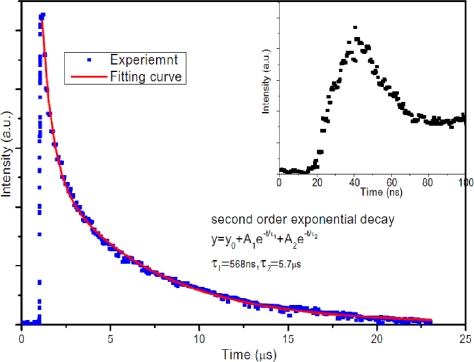
Temporal response of ZnO UV detectors excited by Nd–YAG laser pulses (355 nm, <10 ns). The inset shows the enlarged impulse response [16].

ZnO photoconductors have been investigated in detail by a number of groups. Different materials (Au, Al, Pt, Al/Au, Ni/Au, ITO, *etc.*) have been selected as electrodes [14–26]. Liu *et al.* demonstrated MSM UV sensitive photoconductors based on high-quality N-doped ZnO epitaxial films grown on R-plane sapphire by metal organic chemical vapor deposition (MOCVD) [14]. The low frequency photoresponsivity with the value of 400 A/W at 5 V bias was obtained. The devices show high response speed. The rise time and fall time were about 1 μs and 1.5 μs, respectively. Xu *et al.* have fabricated photoconductive UV detector with planar interdigital Al electrodes on *c*-axis preferred oriented ZnO thin film prepared by RF sputtering on quartz substrate [15]. The detector exhibits fast photoresponse with a rise time of 100 ns and fall time of 1.5 μs. Meanwhile, it shows a big dark current of 38 μA at 5 V bias. We also reported the ZnO UV photoconductor with planar interdigital Au electrodes by RF sputtering [16]. With the applied bias below 3 V, the dark current was below 250 nA. In addition, the transient response measurement revealed fast photoresponse with a rise time of 20 ns as shown in Figure 2. Recently, a back-illuminated vertical-structure ZnO UV detector was fabricated using an indium-tin oxide (ITO) electrode and an Al electrode [17]. At 5 V bias, the dark current was 640 μA and the photocurrent was 16.8 mA under UV illumination (365 nm, 10 μW), indicating the responsivity as high as 1,616 A/W. The response time measurements showed a rise time of 71.2 ns and a decay time of 377 μs.

In order to obtain high performance ZnO photoconductors, different methods such as surface treatment and covering by other materials have been used by several groups [18–20]. The oxygen plasma treatment is found to dramatically enhance the UV detection properties of ZnO, reducing the decay time constant (to below 50 μs) and increasing the on/off ratio of photocurrent (to over 1,000) with high UV responsivity (1–10 A/W) [18]. The reason for this result may be that oxygen plasma treatment can effectively suppress the chemisorption effect and the oxygen vacancy in ZnO films. Additionally, surface HCl treatment [19] and SiO_2_ covering [20] on the surface of devices can increase the photoresponsivity, but they also can increase the dark current due to the damage on ZnO films. More recently, Sun and coworkers reported a photoconductive detector based on intentionally Ga-doped ZnO film on quartz by RF sputtering [27]. The transient response measurement revealed photoresponse with a rise time of 10 ns and a fall time of 960 ns, respectively. The results are much faster than those reported in photoconductive detectors based on unintentionally doped *n*-type ZnO films. And the authors think that the relatively faster response in ZnO:Ga photoconductor may be attributed to the enhancement of tunnel recombination across the potential barriers generated by surface and defects in ZnO:Ga sample, as the similar behavior observed in Si-doped Al_x_Ga_1−x_N photoconductive detectors [28]. Table 1 summarizes the data on responsivity, darkcurrent and response time of ZnO film photoconductors.

### 2.2. MSM Photodiodes

In the past few years, MSM photodiodes have become increasingly popular in the research field due to their fundamental advantages [29,30]:

1. Simple structure.
2. Ease of fabrication and integration.
3. Low capacitance per unit area.

**Figure 3.**
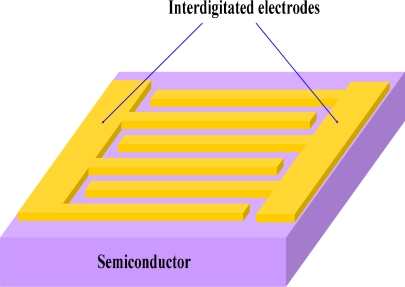
A schematic image of an MSM photodiode structure.

MSM photodiodes are comprised of two back-to-back Schottky diodes by using an interdigitated electrode configuration on top of an active light collection region. A schematic image of the MSM photodiodes structure is shown in Figure 3. This photodetector cannot operate at a zero bias. MSM photodiodes are inherently fast due to their low capacitance per unit area and are usually transit time limited, not RC time constant limited. With electron beam lithography, the electrode width and spacing can be made with submicron dimension which greatly improves the speed. The biggest drawback of MSM photodetectors is their intrinsic low responsivity. MSM detectors exhibit low photoresponsivity mainly because the metallization for the electrodes shadows the active light collecting region.

**Figure 4.**
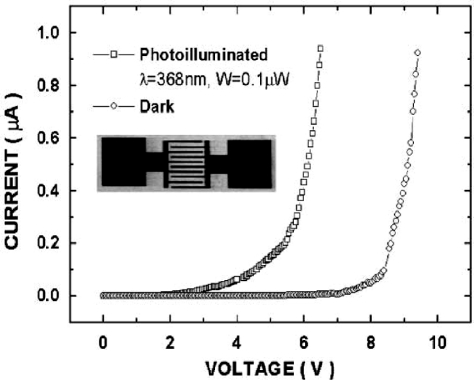
*I–V* characteristics of a ZnO Schottky photodetector with an interdigital structure. The inset shows a SEM picture of the top view of the device [32].

ZnO MSM photodiodes have been fabricated by different methods, such as MOCVD [31,32], laser assisted molecular beam deposition (LAMBD) [33], radio frequency (RF) magnetron sputtering [34–36], atomic-layer deposition (ALD) [37] and molecular beam epitaxy (MBE) [38,39]. In 2001, Liang *et al.* [32] fabricated MSM photodiodes by using Ag as Schottky contact metal. A low frequency photoresponsivity of 1.5 A/W at a bias of 5 V was obtained. The dark current of the device at 5 V bias was in the order of 1 nA (see Figure 4). The photoresponse of the detector showed a fast component with a rise time of 12 ns, and a fall time of 50 ns.

**Figure 5.**
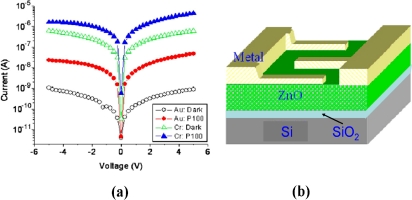
**(a)** Dark and photocurrent characteristics of laser-annealed ZnO MSM-PD structures with Au and Cr metals, **(b)** threed-imensional (3D) cross section of the MSM photodetector structure [33].

**Figure 6.**
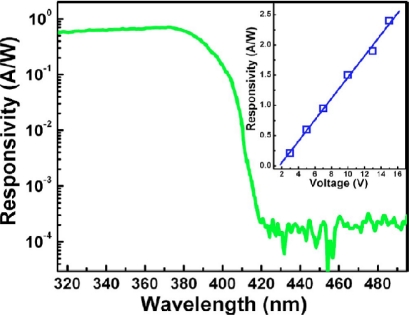
Photoresponse of the photodetector at 5 V bias; the inset shows the dependence of the responsivity of the photodetector at 370 nm on applied bias [37].

After that, MSM photodetectors based on laser-annealed ZnO films with Au and Cr metals were demonstrated by LAMBD [33]. Figure 5 shows the dark and photocurrent characteristics of laser-annealed ZnO MSM photodetectors with Au and Cr metals. The dark current for the Au film was found to be three orders lower in magnitude compared to the Cr film. However, its responsivity was also reduced from 1.05 mA/W to 11.3 μA/W since Au has a higher work function, hence higher barrier height than Cr. Meanwhile, Shan *et al.* have fabricated the ZnO MSM photodiodes by ALD method [37]. The photodetector shows an obvious broad response to the UV spectrum shorter than 400 nm, and the cutoff response wavelength is located at around 390 nm (see Figure 6). By increasing the bias applied, the maximum responsivity of the photodetector increases almost linearly in the range from 3 to 15 V.

According to the results in reference [33], in order to achieve high performance MSM UV photodetectors, it is important to achieve large Schottky barrier height at metal–semiconductor interface. A large barrier height leads to small leakage current and high breakdown voltage, which could result in improved photocurrent to dark current contrast ratio [39]. To achieve a large Schottky barrier height on ZnO, one can choose metals with high work functions [40]. Different metals have been selected as electrode materials, such as Au, Ag, Pt, Ni, Pd, Cr, Al, Ru, *etc.* [31–39]. The Schottky barrier height at the Ru/ZnO, Ag/ZnO, Pd/ZnO and Ni/ZnO was evaluated to be 0.76, 0.736, 0.701 and 0.613 eV, respectively [38,39]. Higher Schottky barrier height can realize lower darkcurrent. However, the responsivity and the quantum efficiency decrease with the increase of the Schottky barrier height.

More recently, Ji *et al.* have demonstrated UV photodetectors based on the ZnO epitaxial films grown on poly(ethylene terephthalate) (PET) flexible substrates by RF sputtering [36]. The device with a stack structure of ZnO/Ag/ZnO/PET served as a Schottky-type MSM photodetector with a ZnO cap layer. The photodetector with a ZnO cap layer shows a much higher UV-to-visible rejection ratio of 1.56 × 10^3^ than that without. This can be attributed to the photocurrents that are not only significantly increased in the UV region but also slightly suppressed in the visible region for such a novel structure. With an incident wavelength of 370 nm and an applied bias of 3 V, the responsivities of both photodetectors with and without a ZnO cap layer are 3.80 × 10^3^ and 2.36 × 10^3^ A/W, which correspond to quantum efficiencies of 1.13 and 0.07%, respectively. However, the photodetector with a ZnO cap layer has larger leakage currents than that without.

### 2.3. Schottky Photodiodes

**Figure 7.**
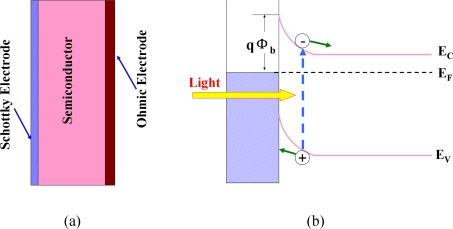
The schematic structure **(a)** and operation **(b)** of the Schottky photoconductor.

Comparing with photoconductor and MSM photodiode, a Schottky photodiode has many advantages in the aspects of high quantum efficiency, high response speed, low dark current, high UV/visible contrast, and possible zero-bias operation [13,41]. The schematic structure and operation of the photoconductor are shown in Figure 7(a,b), respectively. Schottky diodes in their simplest form consist of a metal layer that contacts a semiconductor. The metal/semiconductor junctions exhibit rectifying behavior. The rectifying property of the metal-semiconductor contact arises from the presence of an electrostatic barrier between the metal and the semiconductor which is due to the difference in work functions *Φ_m_* and *Φ_s_* of the metal and semiconductor, respectively. For example, for a metal contact with an *n*-type semiconductor, *Φ_m_* should be greater. For the detail work mechanism of Schottky photodiodes, Razeghi *et al.* have described clearly in Razeghi, *et al.* [13].

In 1986, Fabricius and coworkers first fabricated a ZnO Schottky photodiode using sputtering system [10]. The diode structure consisted of a glass substrate with a Mn electrode as the bottom contact material to the ZnO-Au diode. The diodes exhibited conventional *I-V* characteristics. Due to recombination in the polycrystalline ZnO layers and the contact layers the quantum efficiency was of the order of 1%. Moreover, ZnO Schottky barrier diodes exhibited a rise time around 20 μs and a decay time of 30 μs in the time response measurements. After that, several groups have prepared ZnO Schottky photodiodes by different methods [42–44]. Oh *et al.* have demonstrated ZnO Schottky barrier diodes on (0001) GaN/Al_2_O_3_ substrates by plasma-assisted molecular-beam epitaxy [43]. These ZnO Schottky barrier diodes show a reverse saturation current of ∼10^−8^ A in the dark, and they present a large current buildup of ∼10^3^ A under ultraviolet light illumination, with maintaining stable diode characteristics. The ZnO Schottky barrier diodes have a large bandwidth of 195 nm, where the short-wavelength cutoff and the long-wavelength cutoff are 195 and 390 nm, respectively. Additionally, the devices have a time constant of 0.36 ms.

**Figure 8.**
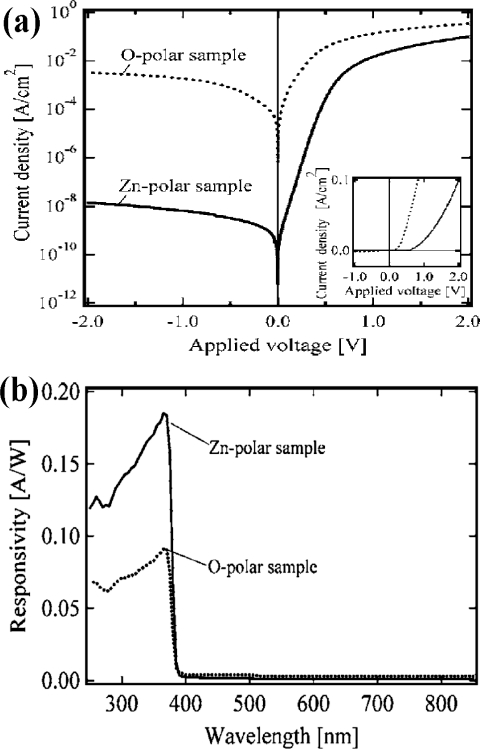
**(a)** Current-voltage characteristics of the Schottky photodiode with a Pt Schottky semi-transparent electrode on the Zn surface (Zn-polar) and O surface (O-polar). **(b)** Spectral responsivity of the photodiodes for the Zn-polar and O-polar devices [44].

In order to investigate the effect of polarization on the response properties, two types of Schottky photodiodes based on Zn-polar and O-polar ZnO films have been fabricated by the hydrothermal growth method [44]. Figure 8(a) shows the typical current-voltage characteristics of the Schottky photodiodes with Pt electrodes. The current increases at 0.8 V with increase in the voltage when the voltage is applied to the Zn-polar sample. On the other hand, in the case of the O-polar sample, the forward biased current increases at 0.4 V, which is smaller than that of the Zn-polar sample. Furthermore, the dark current density of the O-polar device is larger by five orders of magnitude than that of the Zn-polar device. The spectral responses of both Zn-polar and O-polar devices are shown in Figure 8(b). The responsivity and external quantum efficiency for the Zn-polar sample are 0.185 A/W and 62.8%, respectively, at a wavelength of 365 nm, and those for the O-polar sample are 0.09 A/W and 31.0%, respectively. From those measurements, it was found that the responsivity for the Schottky barrier of the Zn surface is two times higher than that for the O surface. The polarity dependence in the Schottky photodiode was attributed to the difference in surface reactivity and/or the defect density in the ZnO substrate surface. These results indicate that it is possible to fabricate high-performance ZnO Schottky photodiodes based on Zn-polar ZnO films.

After that, the ZnO Schottky photodiodes have been demonstrated using transparent polymer as Schottky electrodes [41]. Poly (3,4-ethylenedioxythiophene) poly(styrene sulfonate) (PEDOT:PSS) was selected as electrode material due to the large internal transmittance of nearly 100% in a wide wavelength range from 250 to 800 nm, in addition to a resistivity of as low as 10^−3^ Ω cm and a large work function of 5.0 eV [45]. The quantum efficiency as high as unity in ultraviolet region and a visible rejection ratio of about 10^3^ were achieved in the spectral response of the photodiode under zero-bias condition. The normalized detectivity of the photodiode was evaluated to be 3.6 × 10^14^ cm Hz^1/2^ /W at 370 nm. It is expected that transparent polymers can be used as Schottky electrodes instead of metals for ZnO photodiodes.

More recently, a method to effectively suppress the unwanted increase of the leakage current of ZnO-based Schottky diodes in vacuum was presented by means of a dielectric passivation [42]. Additionally, post-metal deposition annealing has been selected to improve the performance of ZnO based Schottky photodetectors [46]. Ali and coworker found that the performance of the device improves with increasing post-metal deposition annealing temperature up to 250 °C approximately. For annealing temperature beyond 250 °C the performance of the device degrades drastically. The variation in the electrical and photoresponse properties of ZnO based Schottky photodetectors can be attributed to combined effects of interfacial reaction and phase transition during the annealing process. These results suggest that both annealing and dielectric passivation are the viable approach to enhancing the performance of ZnO based Schottky photodetectors.

### 2.4. p-n Junction Photodiodes

**Figure 9.**
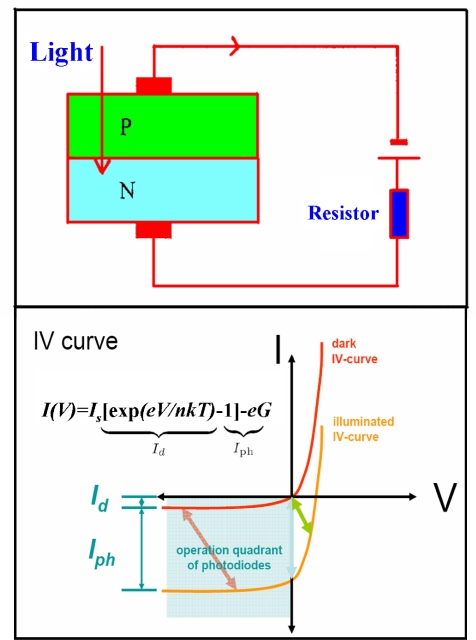
The schematic structure and *I-V* characteristic of *p-n* photodiode.

A *p-n* junction photodiode is just a *p-n* junction diode that has been specifically fabricated and encapsulated to permit light penetration into the vicinity of the metallurgical junction. *p*-*n* and *p*-*i*-*n* photodiodes have the advantages of fast responding speed, low dark current, and working without applied bias. Therefore, *p*-*n* and *p*-*i*-*n* photodiodes are the most suitable choice for future space application. The schematic structure and *I-V* characteristic of *p-n* photodiode are shown in Figure 9. The total current can be expressed as following equation:

(1) $$I(V)=I_{s}\;[\text{exp}(\mathrm{eV}/\mathrm{nkT})-1]-\mathrm{eG}$$

where *I_s_* is the saturation current, *V* is the applied voltage, *n* is the ideality factor, *k* is Boltzmann’s constant, *T* is absolute temperature and *G is* the generation rate. *I_s_* [exp*(eV/nkT)* − 1] and −*eG* correspond to dark current and photocurrent, respectively.

#### 2.4.1. ZnO *p-n* Homojunction Photodiodes

**Figure 10.**
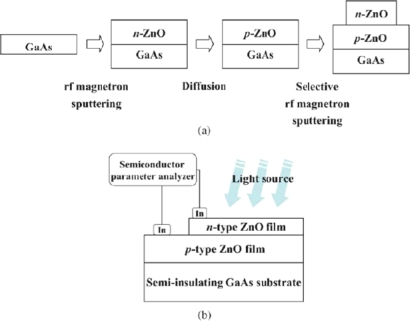
Schematic **(a)** procedure to fabricate a ZnO *p–n* homojunction and **(b)** illustration of a ZnO *p–n* homojunction which had indium electrodes [48].

As is well known, the unintentionally doped ZnO is *n*-type semiconductor for its intrinsic defects, such as oxygen vacancies [47]. A reproducible method to grow *p*-type ZnO film, necessary for fabrication of *p*-*n* junction, is still alluring due to several reasons such as deep acceptor levels, low solubility of the dopants, and the self-compensation process. Therefore, little information can be found about ZnO *p-n* homojunction photodiodes [48–52]. In 2005, Moon *et al.* [48] have fabricated a ZnO *p-n* homojuncion photodiode by RF magnetron sputtering. Fabrication process and schematic structure of a ZnO *p−n* homojunction is schematically illustrated in Figures 10a and 10b, respectively. *P*-type ZnO film was produced by choosing GaAs as a substrate which supplied the dopant element As to ZnO film during a post-annealing in the ambient controlled ampoule. The *p−n* homojunctions exhibited the distinct rectifying current–voltage characteristics. The turn-on voltage was measured to be ∼3.0 V under the forward bias. When UV light (λ = 325 nm) was irradiated on the *p−n* homojunction, photocurrent of ∼2 mA was detected.

**Figure 11.**
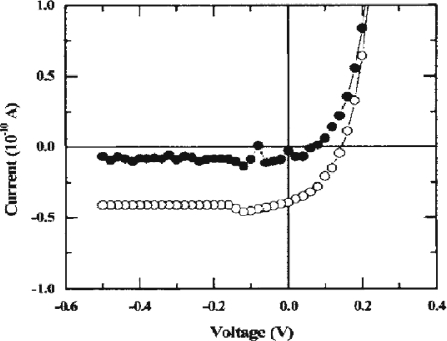
*I-V* characteristics for a ZnO photodiode in the dark and in UV illumination. The curve with solid circles is for the dark current and the curve with open circles for the current under illumination [49].

The same year, ZnO *p*-*n* junctions photodiodes based on As-doped *p*-type ZnO layers with hole concentrations in the mid-10^17^ cm^−3^, and on intrinsic *n*-type ZnO layers with electron concentrations in the mid-10^17^ cm^−3^ were fabricated by hybrid beam deposition (HBD) [49]. The ohmic contacts for ZnO photodiode were formed on each of the *p*-type and *n*-type ZnO surfaces using Ni and Ti. The current-voltage characteristics were measured both in the dark and under UV illumination conditions as shown in Figure 11. The ratio of photo-to-dark current at zero bias is about 20. The dark leakage currents for the ZnO photodiodes are very weak (lower 10^−6^ A/cm^−2^) in the reversed bias configuration. This behavior indicates that ZnO photodiodes might sensitively detect UV light with low noise.

**Figure 12.**
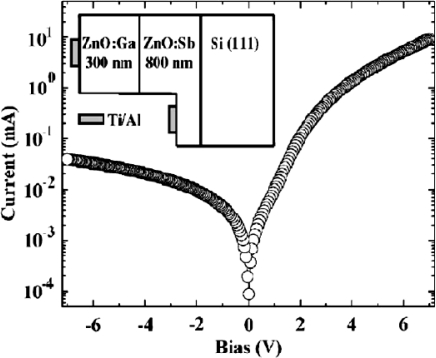
Dark *I-V* curve of a ZnO:Ga/ZnO:Sb homojunction. Absolute value of current is plotted. Inset: Cross-sectional view of the diode. Shaded rectangles represent Ti/Al contacts [51].

After that, ZnO *p*-*n* homojunction photodiodes were fabricated on the ZnO:Ga/ZnO:Sb sample using MBE [50–52]. Al/Ti metal was used to form ohmic contacts on both the *p*-ZnO and *n*-ZnO layers. The rectifying *I*-*V* characteristics show the existence of the ZnO *p*-*n* homojunction and the turn-on voltage is around 2 V (see Figure 12). Very good response to ultraviolet light illumination was observed from photocurrent measurements. Furthermore, forward bias electron injection into the *p* side of a *p*-*n* homojunction could result in an increase of the peak photoresponse and a corresponding increase of the decay constant of the ZnO photodiodes. Both observations are shown to be a consequence of electron trapping [50,51].

#### 2.4.2. ZnO *p-n* Heterojunction Photodiodes

**Figure 13.**
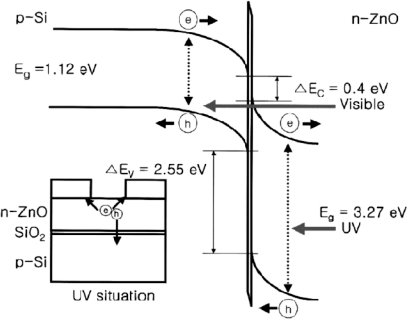
Energy-band diagram of a reverse-biased *n*-ZnO/*p*-Si structure. The small drawing is an illustration showing carrier transports in the depleted *n*-ZnO under UV illumination [57].

**Figure 14.**
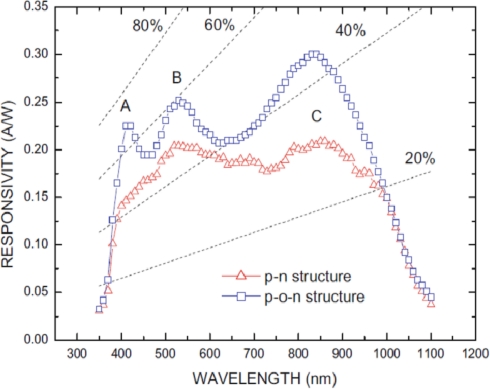
Responsivity as a function of wavelength for a *p*-ZnO/oxide/*n*-Si and *p*-ZnO/*n*-Si structure photodiodes at a bias of −1 V [62].

Owing to the lack of stable and controllable *p*-type ZnO films as mentioned above, in most cases, heterojunctions were used to fabricate ZnO-based UV photodetectors with a different *p*-type semiconductor, such as NiO [53–55], SiC [56], Si [57–68], GaN [69] and so on. Undoubtedly, among all of these *p*-type semiconductors, the commercial silicon have been received much attention for ZnO-based *p*-*n* junction UV photodetectors because of their low cost and widely used integrated circuit technology. In the past few years, n-ZnO/p-Si photodiodes have been fabricated by different groups [57–68]. In 2003, Jeong *et al.* deposited unintentionally doped *n*-ZnO thin films on *p*-type Si substrates by RF magnetron sputtering to form n-ZnO/p-Si photodiodes [57]. A schematic energy band diagram of the relevant *n*-ZnO/*p*-Si heterojunction is shown in Figure 13. The *n*/*p* heterojunction has a thin SiO_2_ layer (3 nm) at the *n*-ZnO/*p*-Si interface and hence the photoelectrons may face a transport barrier. The *n*-ZnO/*p*-Si photodiodes could detect UV photons in the depleted *n*-ZnO and simultaneously detect visible photons in the depleted *p*-Si. However, they show relatively weak response near 380 nm, which is the band gap of ZnO. Furthermore, Chen *et al.* found that an intermediate silicon oxide film can improve the quantum efficiency and the responsivity by decreasing the surface state density and increase the tunneling photocurrent [62]. Figure 14 plots the responsivity as a function of wavelength for both a *p*-ZnO/*n*-Si and a *p*-ZnO/oxide/*n*-Si photodiode, measured throughout this work at a reverse bias of 1 V. The responsivity of the photodiodes exhibited three distinct regions of behaviors around wavelengths of 400 nm, 530 nm, and 850 nm, denoted regions A, B, and C, respectively. In the *p*-ZnO/oxide/*n*-Si structure, in regions A, B, and C, the responsivity was 0.225, 0.252 and 0.297 A/W, respectively. As for the *p*-ZnO/*n*-Si structure, however, in regions A, B, and C, the responsivity was 0.147, 0.204 and 0.206 A/W, respectively.

**Figure 15.**
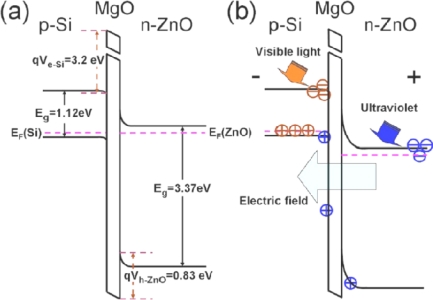
The energy-band diagrams of *n*-ZnO/insulator-MgO/ *p*-Si heterojunctions **(a)** under zero bias and in dark and **(b)** under reverse bias and in light illumination. The circles with a line and a cross inside stand for the photogenerated electrons and holes, while the brown and blue relate to the visible and UV excitation in depletion regions of *p*-Si and *n*-ZnO, respectively [65].

According to the above introduction, it seems that *n*-ZnO/*p*-Si heterostructures are very suitable for UV photodetectors. However, the *n*-ZnO/*p*-Si photodetectors retain an obvious photoresponse to visible light, although the UV photoresponse is increased due to ZnO, which would limit its direct application in UV detection under a visible light background. In order to realize visible blind UV photodetectors, Zhang *et al.* have fabricated a photodetector based on a double heterojunction of *n*-ZnO/insulator-MgO/ *p*-Si grown by MBE [65]. The photodetector shows a rectification ratio of ∼10^4^ at ±2 V and a dark current of 0.5 nA at a reverse bias of −2 V. The photoresponse spectrum indicates a visible-blind UV detectivity of the devices with a sharp cut off at the wavelength of 378 nm and a high UV/visible rejection ratio. The energy band diagram of the *n*-ZnO/insulator-MgO/ *p*-Si double heterojunction derived from Anderson model is drawn in Figure 15. Visible light can transmit through the *n*-ZnO film and be absorbed in the depletion region of *p*-Si, resulting in the photogeneration process of electron-hole pairs. The internal electric field drives the photogenerated electrons toward the *n*-ZnO side, but they cannot cross over the interface between *p*-Si and *i*-MgO due to the high potential barrier (3.2 V) for electrons and immediately recombine with holes, which results in the block of the consecutive photogenerated process. That is the reason why no visible response was observed. On the other hand, UV light with a wavelength shorter than 378 nm is absorbed in the depletion region of *n*-ZnO, which results in photogenerated electron-hole pairs. The middle *i*-MgO layer takes the dual role, *i.e.*, a buffer layer for the epitaxial growth of the *p*-insulator-*n* double heterojunction and a barrier layer for the realization of visible-blind UV detectivity of the *p*-insulator-*n* photodetector with a high UV/visible rejection ratio. Their work indicated that an obvious suppression of photoresponse to visible light can be realized by MgO interlayer for *n*-ZnO/ *p*-Si photodetectors.

Additionally, Chen *et al.* reported that with a coating of monolayer silica nanoparticles on the surface of n-ZnO/p-Si photodiode, the photoresponsivity at wavelengths between 400 and 650 nm is enhanced by an average of 17.6% [64]. The authors think that the increase of the photoresponsivity is due to the improved optical transmission toward the semiconductor through the silica nanoparticles. Furthermore, the acceptance angle of the nanoparticle coated device between 400 and 650 nm is dramatically increased, which is attributed to the effect of Bragg diffraction.

Besides *p*-type silicon, ZnO *p-n* heterojunction photodiodes based on wide band gap *p*-type semiconductors such as NiO [53–55], SiC [56] and GaN [69] have also been reported by many groups. Because both two semiconductor layers in *p-n* heterojunction photodiodes are visible transparent, these devices are intrinsic visible-blind UV photodetectors. Ohta *et al.* found that efficient UV-response for *n*-ZnO/*p*-NiO photodiode was observed up to ∼0.3 A/W at 360 nm (−6 V biased) [53,54]. Meanwhile, Alivov *et al.* have demonstrated a *n*-ZnO/*p*-SiC heterojunction photodiode made by MBE [56]. Current-voltage characteristics of the structures had a very good rectifying diode-like behavior with a leakage current less than 2 × 10^−4^ A/cm^2^ at −10 V, a breakdown voltage greater than 20 V, a forward turn on voltage of ∼5 V, and a forward current of ∼2 A/cm^2^ at 8 V. A photoresponsivity of as high as 0.045 A/W at −7.5 V reverse bias was observed for photon energies higher than 3.0 eV. More recently, Zhu *et al.* have deposited undoped *n*-type ZnO film on *p*-type GaN substrate to form a *p-n* heterojunction photodiode using MBE [69]. Under back-illumination conditions, the photodetector shows an enhanced UV photoresponse in a narrow spectrum range of only 17 nm in width. The authors attributed this high selectivity to the GaN layer that acts as a “filter” for the photodetector.

Here, we tabulate the representative results on photodetector properties of ZnO *p-n* heterojunction photodiodes reported so far, along with a brief description of the corresponding device continuations, detection wavelength, and photodetector performance (Table 2).

## 3. ZnMgO-Based Photodetectors

The UV region is commonly divided into the following subdivisions with different wavelength regions:

$$\begin{array}{c} \text{UV}-A\;\;\;\;400-320\;\text{nm}\\ \text{UV}-\text{B}\;\;\;\;320-280\;\text{nm}\\ \text{UV}-\text{C}\;\;\;280-200\;\text{nm}\\ \text{Vacuum}\;\text{UV}\;\;\;200-10\;\text{nm} \end{array}$$

As is well known, the sun is a strong source of UV radiation. Due to the ozone layer absorption, the high-energy solar photons with wavelengths shorter than ∼280 nm can not reach the earth, which is refereed as solar-blind region [70]. Photodetectors which respond only to radiation with λ < 280 nm are defined as solar-blind photodetectors. Within the atmosphere, due to the lack of solar radiation background, if a solar-blind photodetector detects a signal, it should originate from an external UV emitter (flame, missile plume, *etc.*) [13]. Therefore, one particular application of solar-blind UV photodetectors is the missile threat warning system.

Solar-blind UV flame detectors are typically based on wide-bandgap semiconductors such as MgZnS [71], AlGaN [72], diamond [73], Ga_2_O_3_ [74], LaAlO_3_ [75] and ZnMgO [76–80]. Among them, ZnMgO material system possesses unique figures of merit, such as large tunable band-gap energy (3.3–7.8 eV) [81–83] and strong radiation hardness [84]. From the view of materials and devices fabrication, relatively low growth temperatures (100–750 °C) can ease the thin film epitaxial growth [83] and many techniques such as MBE [85], MOCVD [77–79], PLD [81–83], RF sputtering [86–88] have been proven to be successful in achieving high quality films. The availability of lattice-matched single-crystal substrates (ZnO and MgO for hexagonal and cubic ZnMgO films, respectively) is another advantage for ZnMgO fabrication [89]. Additionally, ZnMgO is an environment friendly material. Therefore, ZnMgO should be an excellent choice for optoelectronic devices in the ultraviolet portion of the spectrum.

### 3.1. ZnMgO Photoconductors

**Figure 16.**
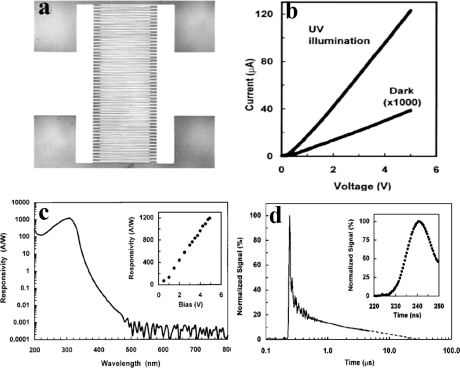
**(a)** Optical microscope picture of a Mg_0.34_Zn_0.66_O UV detector with MSM structure. **(b)** *I*-*V* curves show dark current and photocurrent under 308 nm, 0.1 μW UV light illumination. **(c)** Spectral response of a Mg_0.34_Zn_0.66_O UV detector biased at 5 V. **(d)** Temporal response of Mg_0.34_Zn_0.66_O UV detectors excited by nitrogen gas laser pulses (337.1 nm, <4 ns) [90].

In 2001, Yang *et al.* reported the fabrication of ZnMgO photoconductor by PLD and investigated the photoconductive properties [90]. Mg_0.34_Zn_0.66_O thin films with a band gap of 4.05 eV were epitaxially grown on *c*-plane sapphire substrates. Based on the Mg_0.34_Zn_0.66_O films, planar geometry photoconductive type metal–semiconductor–metal photodetectors were fabricated. The interdigital metal electrodes, which were defined on ∼1500 Å Cr/Au bilayer by conventional photolithography and ion milling, are 250 μm long, 5 μm wide, and have an interelectrode spacing of 5 μm [see Figure 16(a)]. Figure 16b shows the linear *I*–*V* curves both in dark and under 308 nm light illumination. Under 5 V bias, the measured average dark current is ∼40 nA, which is close to the calculated dark current based on the resistivity of Mg_0.34_Zn_0.66_O. Upon UV illumination (308 nm, 0.1 μW), the photocurrent jumped to 124 μA at 5 V bias, indicating a responsivity of ∼1,200 A/W. This responsivity value is comparable to that of ZnO (400 A/W at 5 V bias, 2–16 μm interelectrode spacing) and GaN (2,000 A/W at 5 V bias, 10 μm interelectrode spacing) photoconductive detectors [14,91]. The spectral response of a Mg_0.34_Zn_0.66_O UV detector under front illumination is plotted in Figure 16(c). The peak response is found at 308 nm. The cutoff wavelength is ∼317 nm, and the visible rejection (R308 nm/R400 nm) is more than four orders of magnitude, indicating a high degree of visible blindness. Figure 16d shows the temporal response of a Mg_0.34_Zn_0.66_O UV detector with 3 V bias and 50 V load. The 10%–90% rise and fall time are 8 ns and ∼1.4 μs, respectively. The excess lifetime of trapped carriers, especially the trapped holes in *n*-type semiconductors should be responsible for the slow decay process [90]. After that, Ghosh *et al.* have fabricated Mg_x_Zn_1−x_O (x = 0 − 0.08) thin films on glass substrate by sol-gel technique and photoconductivity of the thin films have been investigated in vacuum, hydrogen, oxygen and air [92]. The authors found that the I_ph_/I_d_ for the Mg_x_Zn_1−x_O films increases in vacuum, hydrogen with increase in *x*, the increase being maximum in vacuum for the film with x = 0.05. The I_ph_/I_d_ does not change much for oxygen and it remains almost constant for air. The photoresponse is much slower in the vacuum, hydrogen and oxygen ambient compared to that in air.

### 3.2. ZnMgO MSM Photodiodes

**Figure 17.**
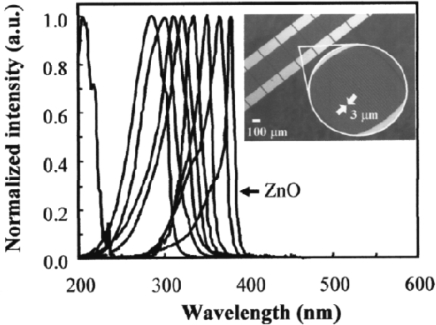
Normalized spectral response of an array of UV photodetectors based on a composition spread of Mg_x_Zn_1−x_O. The active area of each device was 250 × 220 μm^2^. Composition variation within each detector is less than 2.4 mol %. The inset of Figure 18 shows an enlarged picture of interdigited electrodes used as detectors. Each finger width and the finger separation is 3 mm [94].

In 2003, Yang and coworkers reported ZnMgO MSM UV photodiodes [93]. Wide-band-gap cubic-phase MgZnO thin films were grown on Si (100) with a thin SrTiO_3_ buffer layer by PLD [93]. Photodetectors fabricated on Mg_0.68_Zn_0.32_O/SrTiO_3_/Si show peak photoresponse at 225 nm, which is in the deep UV region. At the same time, Takeuchi *et al.* have fabricated Mg_x_Zn_1−x_O epitaxial composition spreads where the composition across the chip is linearly varied from ZnO to MgO [94]. The continuously changing band gap across the spread is used as a basis for compact broadband photodetector arrays with a range of detection wavelengths separately active at different locations on the spread film. The composition-spread photodetector is demonstrated in the wavelength range of 290–380 nm using the ZnO to Mg_0.4_Zn_0.6_O region of the spread as shown in Figure 17.

**Figure 18.**
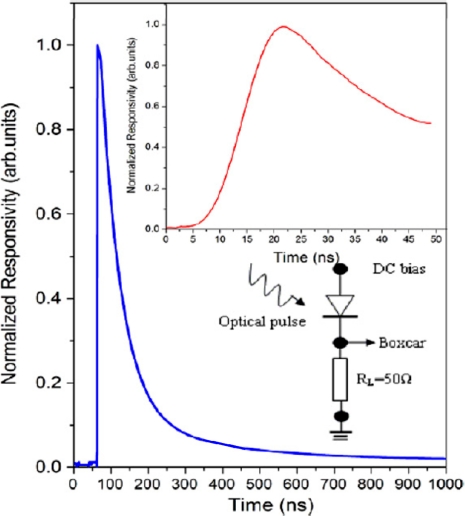
Normalized pulse response measurement of the Zn_0.8_Mg_0.2_O UV detector excited by Nd-YAG laser pulses (266 nm, ∼10 ns) [86].

**Figure 19.**
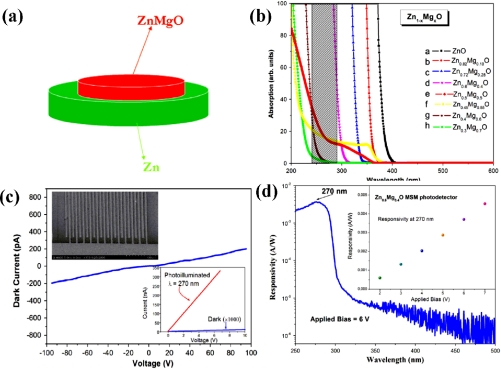
**(a)** The schematic diagram of the composite ZnMgO–Zn target. **(b)** The UV–visible absorption spectra of Zn_1−x_Mg_x_O films with different *x* values. **(c)** The typical *I*–*V* characteristics of the Zn_0.6_Mg_0.4_O film based MSM detector **(d)** The spectral response of the Zn_0.6_Mg_0.4_O UV detector with a 5 μm finger pitch biased at 6V. The inset shows the responsivity at 270 nm as a function of reverse bias [88].

After that, several groups have reported the production of ZnMgO MSM photodiodes by different methods, such as RF sputtering [86–88,95], MBE [76,96,97], MOCVD [77–79], Sol-Gel [98,99] and PLD [100]. Zn_0.8_Mg_0.2_O MSM UV photodiodes were fabricated on quartz by RF sputtering [86]. The photodetectors showed a peak responsivity at 330 nm. The ultraviolet-visible rejection ratio (*R*330 nm/*R*400 nm) was more than four orders of magnitude at 3 V bias. The photodetector showed fast photoresponse with a rise time of 10 ns and a fall time of 170 ns (see Figure 18). The thermally limited detectivity was calculated to be 3.1 × 10^11^ cm Hz^1^*^/^*^2^W^−1^ at 330 nm. In addition, a composite target was selected to prepare ZnMgO films by RF magnetron sputtering and the Mg composition of the samples can be controlled easily, even at a high growth temperature [88]. The schematic diagram of the composite ZnMgO–Zn target was shown in Figure 19a. Figure 19b shows the UV–visible absorption spectra of Zn_1−x_Mg_x_O films with different *x* values. The absorption edges of pure ZnO, Zn_0.82_Mg_0.18_O, Zn_0.72_Mg_0.28_O, Zn_0.6_Mg_0.4_O, Zn_0.4_Mg_0.6_O and Zn_0.3_Mg_0.7_O were at about 385 nm, 370 nm, 330 nm, 295 nm, 240 nm and 225 nm, respectively. The phase separation was evident for compositions whose absorption edge was between 290 and 240 nm. The typical *I*–*V* characteristics of the Zn_0.6_Mg_0.4_O film based MSM detector were shown in Figure 19c. The device exhibited a very low dark current (lower than 200 pA for |*V*_bias_| *<* 95 V), and it was not broken down even when the applied bias voltage is larger than 100 V. Furthermore, the MSM deep ultraviolet photodetector based on the wurtzite Zn_0.6_Mg_0.4_O film exhibits a peak responsivity at 270 nm and a very sharp cutoff wavelength at around 295 nm [see Figure 19(d)]. Recently, cubic Mg_0.70_Zn_0.30_O thin films have been prepared on quartz substrates by RF magnetron sputtering, and an MSM structured photodetector was fabricated based on the film [95]. The peak responsivity of the photodetector was at about 225 nm, with a very sharp cutoff wavelength at about 230 nm. The dark current of the photodetector was only 2 pA at 3 V bias. These results indicated that RF sputtering system can be used to fabricate high performance solar-blind UV photodetectors.

Joike *et al.* have fabricated single-phase wurtzite Zn_1−x_Mg_x_O alloy films with 0 < x < 0.45 on (111)-oriented Si substrates by MBE [97]. The cutoff wavelength of Zn_1−x_Mg_x_O UV photodetectors with the x values of 0, 0.10, 0.26 and 0.34 is at ∼375, ∼350, ∼315 and ∼300 nm, respectively. Du and coworkers have demonstrated that the interfacial layer plays a key role in suppressing phase segregation in the MgZnO layer [76]. A single-phase wurtzite Mg_0.55_Zn_0.45_O thin film with a bandgap of 4.55 eV was successfully synthesized on quasi-homo Mg_0.2_Zn_0.8_O buffers by RF-plasma assisted MBE. The photodetector based on Mg_0.55_Zn_0.45_O thin film shows a sharp cut off of responsivity at 277 nm and the responsivity peak is at 266 nm, which demonstrates that the device is a real solar-blind photodetector.

More recently, ZnMgO MSM photodetectors have also been fabricated by MOCVD. Ju *et al.* have prepared the MgZnO thin films with Mg content from 0.5 to 0.7 by MOCVD [77]. A series of solar blind UV photodetectors with their cutoff wavelength varying from 225 to 287 nm has been realized based on these thin films. The representative solar-blind photodetector shows a UV/visible rejection ratio of about four orders of magnitude and a dark current of 15 pA under 10 V bias.

### 3.3. ZnMgO Schottky Photodiodes

In 2008, Endo *et al.* reported the fabrication and characteristics of Pt/Mg_x_Zn_1−x_O Schottky photodiodes on a ZnO Single Crystal [101,102]. The Mg_x_Zn_1−x_O film was deposited on a ZnO single crystal substrate by RF magnetron sputtering method. The optical bandgap of the Mg_0.59_Zn_0.41_O film obtained from the spectral transmittance and reflectance was 4.6 eV. The fabricated photodiode consisted of an anti-reflection SiO_2_ film, semitransparent Schottky Pt electrode, Mg_0.59_Zn_0.41_O film, *n^+^*-ZnO single crystal substrate and Pt/Ti ohmic electrode. The ideality factor of the photodiode, obtained from the current–voltage characteristics, was 1.3. The maximum responsivity was 0.015 A/W at the wavelength of 220 nm. After that, Nakano *et al.* demonstrated the Schottky photodiodes consisting of a Mg_x_Zn_1−x_O (x ≤ 0.43) thin film and a transparent conducting polymer, poly (3,4-ethylenedioxythiophene) poly(styrenesulfonate) by MBE [103]. Spectral response of Mg_x_Zn_1−x_O Schottky photodiodes was characterized under a zero-bias condition at room temperature. The cut-off wavelengths could be varied systematically by changing x, keeping high quantum efficiency near unity and small Urbach’s energy up to sufficiently high Mg content (x ≤ 0.43). More recently, Schottky photodiodes based on Au-ZnMgO/sapphire are demonstrated covering the spectral region from 3.35 to 3.48 eV, with UV/VIS rejection ratios up to ∼10^5^ and responsivity as high as 185 A/W [104]. Both the rejection ratio and the responsivity are shown to be largely enhanced by the presence of an internal gain mechanism. The authors think that during illumination the Schottky photodiodes become highly ohmic due to the large contribution of tunneling, whose primary origin is the photoexcitation of trapped carriers at acceptor-like deep levels. This tunneling current causes a large internal gain under reverse bias, which is a function of the photon flux. At the same time, Zhu *et al.* have demonstrated a Au/MgO/MgZnO metal-oxide-semiconductor-structured photodetector by MOCVD [105]. The responsivity of the photodetector was about two orders of magnitude larger than that of the Au/MgZnO metal-semiconductorstructured photodetector fabricated under the same procedure except that no MgO layer was introduced. The detectivity of the photodetector can reach 1.26 × 10^13^ cm Hz^1/2^/W, almost one order of magnitude larger than that of Si photodetectors that are widely employed for UV detection currently. The authors think that the enhanced responsivity should be attributed to the carrier multiplication occurring in the MgO layer via impact ionization. Their results may provide a facile route to ultraviolet photodetectors with high internal gain.

### 3.4 ZnMgO p-n Junction Photodiodes

**Figure 20.**
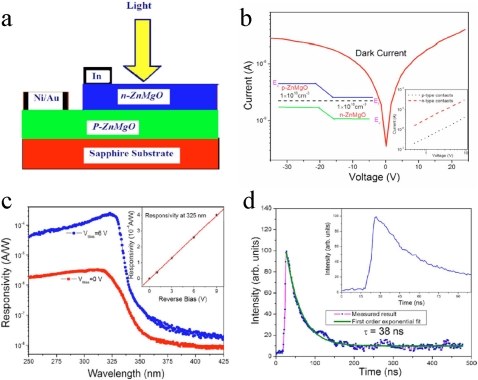
**(a)** Schematic diagram of the Zn_0.76_Mg_0.24_O *p*-*n* photodiode. **(b)** *I*-*V* plot of the homojunction diode in dark. The left inset gives the energy band diagram at equilibrium. The right inset gives the *I*-*V* plots of the *p*- and *n*-type ohmic contacts. **(c)** Spectral response of the Zn_0.76_Mg_0.24_O *p*-*n* photodiode with the reverse bias of 0 and 6 V. The inset shows the responsivity at 325 nm as a function of reverse bias. **(d)** The pulse response measurement on the Zn_0.76_Mg_0.24_O *p*-*n* photodiode excited by Nd:YAG pulsed laser with a 50 Ω impedance [85].

Just as mentioned above, it is still a challenge to fabricate reliable *p*-type ZnO or ZnMgO. Therefore, very few reports can be found about ZnMgO *p-n* homojunction photodiodes [85,106]. In 2007, the first Zn_0.76_Mg_0.24_O *p-n* homojunction photodiode has been prepared on (0001) Al_2_O_3_ substrate by MBE [85]. Ni/Au and In metals deposited using vacuum evaporation were used as *p*-type and *n*-type contacts, respectively. The schematic structure of the device was shown in Figure 20(a). Current-voltage measurements on the device showed weak rectifying behavior [see Figure 20(b)]. Both *p* and *n* electrodes are good ohmic contacts. This result indicates that the rectifying behavior comes from the *p*-*n* junction instead of the metal-semiconductor contacts. In Figure 20(c), the response spectra indicated that peak responsivity of the device is at around 325 nm. The ultraviolet-visible rejection ratio (*R*325 nm/*R*400 nm) of four orders of magnitude was obtained at 6 V bias. The photodetector showed fast photoresponse with a rise time of 10 ns and fall time of 150 ns as shown in Figure 20(d). In addition, the thermally limited detectivity was calculated as 1.8 × 10^10^ cm Hz^1/2^/W at 325 nm, which corresponds to a noise equivalent power of 8.4 × 10^−12^ W/Hz^1/2^ at room temperature.

After that, Shukla reported on Zn_1−x_Mg_x_O homojunction photodetectors on (0001) sapphire substrate fabricated by a PLD system [106]. Ti-Au and Ni-Au metals deposited using vacuum evaporation were used as *n*-type and *p*-type contacts, respectively. The dark current of the Zn_1−x_Mg_x_O photodetectors are smaller than 20 pA at the bias voltage of 10 V and smaller than 2 nA at a bias less than 40 V. The cutoff wave length of the Zn_1−x_Mg_x_O photodetectors varied from 380 nm to 284 nm and the corresponding rejection decreased from 886 to 842 with the increase of Mg content (x) from 0 to 0.34. The decrease of rejection ratio can be attributed to the degradation in the crystallinity of the Zn_1−x_Mg_x_O films due to the doping to Mg. Meanwhile, Li and coworkers demonstrated *p*-Mg_0.2_Zn_0.8_O/*n*-ZnO heterojunction ultraviolet photodiode on a sapphire substrate by MBE [96]. The current–voltage measurement indicates that the heterojunction has a weak rectifying behaviour with a turn-on voltage of ∼5 V. The spectral response measurement shows that the photodiode has a peak responsivity at around 340 nm, and it has a wide detection range in the ultraviolet region from 400 to 320 nm. The response in the long and short wavelength region is due to the contribution of the *n*-ZnO and *p*-MgZnO layers, respectively. The ultraviolet–visible rejection ratio (*R*340 nm/*R*500 nm) of two orders of magnitude was obtained at a reverse bias of 8 V. More recently, the spectral response of back- and front-surface-illumination MgZnO/ZnO *p-n* ultraviolet photodetector have been investigated [107]. The peak responsivity at 330 nm for the device under back-illumination is about four times larger than that of the device under front-illumination under the same reverse bias. Comparing with front-illumination, enhancement in peak responsivity at 330 nm under back-illumination was achieved for the reduction of the surface recombination velocity.

Additionally, the single-phased wurtzite ZnO based oxide system Zn_1−x−y_Be_x_Mg_y_O had been successfully prepared with a bandgap continuously modulated from 3.7 to 4.9 eV, and the advantage of crystalline and optical properties had been demonstrated compared with those of ZnMgO and BeZnO [108]. Therefore, it is expected that Zn_1−x−y_Be_x_Mg_y_O based UV photodetectors may have high performance. Considering the difficulty of achieving stable and reproducible high-quality *p*-type ZnO layers, zero-biased solar-blind photodetectors based on the *n*-Zn_1−x−y_Be_x_Mg_y_O/*p*-Si heterojunction with a cutoff wavelength of 280 nm were fabricated by PLD [109]. The responsivity of the device was 0.003 A/W at zero bias with a UV/visible rejection ratio of more than two orders of magnitude. Furthermore, the improvement in responsivity was achieved by enhancing the carrier collection efficiency using a thin Al-doped ZnO contact layer, and the peak responsivity significantly improved to 0.11 A/W at zero bias, corresponding to a high external quantum efficiency of 53%. The rise time achieved was as fast as 20 ns. Although the fall time was 250 μs, it could be potentially shortened by preparing the contact layer with higher crystalline quality. This work demonstrates the possibility of a wurtzite ZnBeMgO oxide system in realizing high performance zero-biased photodetector with a typical solar-blind photoelectric response characteristic.

## 4. Conclusion and Perspectives

During the past few tears, impressive research efforts have been concentrated on the fabrication and performance of ZnO-based UV photodetectors. Recently, ZnO-based UV photodetectors show good responsivity, high UV/visible contrast ratio, high speed and low noise characteristics, and show the tunability of the detection edge from 380 nm to 225 nm just by varying the Mg mole fraction. All these results indicate that RF sputtering, PLD, MBE and MOCVD are very suitable methods to fabricate ZnO-based UV photodetectors, especially for ZnMgO solar-blind photodetectors. However, the technology of ZnO-based materials and photodetectors is not yet mature. This should inspire more research efforts to address the challenges that remain, as noted below:

1. *The fabrication of p-type ZnO-based materials:* The *p*-type doping for ZnO-based materials has been recognized as a major obstacle for achieving high performance UV photodetectors. As is well known, *p-n* junction photodetectors have the advantage of high sensitivity, fast responding speed and low dark current. However, the lack of stable and reproducible *p*-type ZnO-based materials hinders the *p-n* junction photodetectors. Although comprehensive efforts have been made towards the synthesis of high-quality *p*-type ZnO-based materials and several ZnO-based *p-n* homojunction photodetectors have been reported, significant challenges still exist in their syntheses and qualities that include, but not limited to, the stability, reproducibility and the control of carrier concentration and mobility for *p*-type ZnO-based materials.
2. *The fabrication of high quality Zn_1_*_−_*_x_Mg_x_O films:* In order to realize Zn_1−x_Mg_x_O films photodetectors with different detective wavelengths, the fabrication of high quality Zn_1−x_Mg_x_O films with different x values is necessary. However, a wide miscibility gap exists in the ZnO–MgO binary system due to the structure difference and large lattice mismatch between ZnO (wurtzite, 3.25 Å) and MgO (rock salt, 4.22 Å). Therefore, the phase segregation and the low crystal quality has become the key problem for fabricating MgZnO films.
3. *The relatively slower response speed:* ZnO-based photodetectors, especially for ZnO-based photoconductors usually have a slow response speed due to the adsorption and dis-adsorption of oxygen molecular near the surface of semiconductors. In order to meet the future demands in variety of fields, the response speed for ZnO-based photodetectors must be improved.

## Figures and Tables

**Table 1.** Comparison of the ZnO photoconductors performance.

	**Fabrication Method**	**Electrodes**	**Doping or Treating**	**Dark current**	**Responsivity**	**Response time**	**Ref.**
**photoconductors based on ZnO films**	PLD	Al	____	0.2 mA/5 V	____	50 s (rise time) 120 s (fall time)	[4]
	MOCVD	Al	N-doping	450 nA/5 V	400 A/W at 5 V bias	1 μs (rise time) 1.5 μs (fall time)	[14]
	RF Sputtering	Al	____	38 μA / 5 V	18 A/W at 5 V bias	100 ns (rise time) 1.5 μs (fall time)	[15]
	RF Sputtering	Au	____	250 nA / 3 V	30 A/W at 3 V bias	20 ns (rise time) 10 μs (fall time)	[16]
	RF Sputtering	Al, ITO	____	640 μA / 5 V	1616 A/W at 5 V bias	71.2 ns (rise time) 377 μs (fall time)	[17]
	RF Sputtering	Al	Oxygen plasma treatment	400 pA/3 V	1–10 A/W	50 μs (fall time)	[18]
	MBE	Ni/Au	____	38 mA/ (100 V/cm)	<0.05 A/W	0.556 ms (fall time)	[22]
			HCl treatment	0.1–0.2 mA/4 V	0.141 A/W at 10 V bias	____	[19]
	P-MBE	Al/Ti	Ga-doping	10 mA/5 V	1.68 A/W at 20 V bias	95 s (rise time) 2068 s (fall time)	[23]
	Sol-gel	Au	____	∼8 mA/1.5 V	∼0.03 A/W at 5 V bias	160 s (drop to 50% of its maximum Value)	[24]
	RF Sputtering	Al	Ga-doping	____	2.6 A/W at 10 V bias	10 ns (rise time) 960 ns (fall time)	[27]

**Table 2.** ZnO-based *p-n* heterojunction photodiodes.

**Device structure**	**Fabrication Method**	**Electrodes**	**Detecting range**	**Forward threshold voltage**	**Dark current**	**Responsivity**	**Response time**	**Ref.**
ZnO/NiO:Li	PLD	Au; ITO	UV	1 V	____	0.3 A/W at −6 V bias (360 nm)	____	[53,54]
*p*-NiO/ *i*-ZnO/*n*-ITO	e-beam evaporation	____	UV	1 V	10 nA/cm^2^ (−5 V)	____	____	[55]
*n*-ITO/ *i*-ZnO/ *p*-NiO				2 V	100 nA/cm^2^ (−5 V)			
*n*-ZnO/*p*-SiC	MBE	Au/Al; Au/Ni	UV	5 V	2×10^−4^ A/cm^2^ (−10 V)	0.045 A/W at −7.5 V bias	____	[56]
*n*-ZnO/*p*-Si	RF Sputtering	Au/Al; In	UV/Visible	____	____	0.5 A/W (310 nm) and 0.3 A/W (650–nm) at −30 V bias	____	[57]
*n*-ZnO/*p*-Si	sol-gel	Au	UV/Visible	1 V	7.6×10^−5^ A/cm^2^ (−5 V)	____	____	[58]
*n*-ZnO/*p*-Si	RF Sputtering	In; Cu	UV/Visible	____	∼10^−4^ − 10^−3^ A/cm^2^ (−5 V)	0.14–0.29 A/W at −5 V bias	35 ns	[59,60]
ZnO:Al/*p*-Si	Sol-gel	Au	UV/Visible	____	____	0.22 A/W at −5 V bias (420 nm)	____	[61]
*n*-ZnO/SiO2/*p*-Si	Ultrasonic Spray pyrolysis	Ni/Au; Ti/Pt/Au	UV/Visible	____	4.98×10^−10^ A (−1 V)	0.225–0.297 A/W at −1 V bias	____	[62]
*Si particles coated n*-ZnO/*p*-Si	RF Sputtering	Ni/Au; Ti/Au	UV/Visible	∼4 V	4.7×10^−6^ A/cm^2^ (−3 V)	____	____	[64]
*n-*ZnO/i-MgO/ *p*-Si	MBE	Ti/Au;In	UV	∼1.5 V	<1 nA (−2 V)	____	____	[65]
*AlO coated n*-ZnO/*p*-Si	RF Sputtering	Au-Al; In	UV/Visible	____	____	0.06 A/W at −5 V bias (310 nm)	____	[66]
*n*-ZnO/*p*-Si	RF Sputtering	Au-Al	UV/Visible	____	____	0.35 A/W at −5 V bias (650 nm)	____	[67]
*Ni/n*-ZnO/*p*-Si	DC magnetron sputtering	Ni	UV/Visible	____	1 μA (−8 V)	210 A/W (390 nm) and 110 A/W (850 nm) at −5 V bias	10^−7^ s	[68]
*n*-ZnO/*p*-GaN	MBE	Ni/Au; In	UV	4.6 V	____	∼10^−6^ A/W (370 nm) at 0 V bias	____	[69]
